# An alternative data filling approach for prediction of missing data in soft sets (ADFIS)

**DOI:** 10.1186/s40064-016-2797-x

**Published:** 2016-08-15

**Authors:** Muhammad Sadiq Khan, Mohammed Ali Al-Garadi, Ainuddin Wahid Abdul Wahab, Tutut Herawan

**Affiliations:** Department of Information Systems, Faculty of Computer Science and Information Technology, University of Malaya, Kuala Lumpur, Malaysia

**Keywords:** Soft sets, Data filling, Decision making, Incomplete information systems, Parameters association

## Abstract

Soft set theory is a mathematical approach that provides solution for dealing with uncertain data. As a standard soft set, it can be represented as a Boolean-valued information system, and hence it has been used in hundreds of useful applications. Meanwhile, these applications become worthless if the Boolean information system contains missing data due to error, security or mishandling. Few researches exist that focused on handling partially incomplete soft set and none of them has high accuracy rate in prediction performance of handling missing data. It is shown that the data filling approach for incomplete soft set (DFIS) has the best performance among all previous approaches. However, in reviewing DFIS, accuracy is still its main problem. In this paper, we propose an alternative data filling approach for prediction of missing data in soft sets, namely ADFIS. The novelty of ADFIS is that, unlike the previous approach that used probability, we focus more on reliability of association among parameters in soft set. Experimental results on small, 04 UCI benchmark data and causality workbench lung cancer (LUCAP2) data shows that ADFIS performs better accuracy as compared to DFIS.

## Background

Soft set theory proposed by Molodtsov is considered as a mathematical model for dealing with vague and uncertain data (Molodtsov 1999). This theory is a standard as compare to existing theories such as fuzzy set, rough set, vague set and statistical approach for dealing with vague data because of its adequate of parameterization. Research in the soft set theory both theoretical and practical has been attracted many attentions, especially in the field of decision making. The first attempt in soft set decision making is introduced by Maji et al. (2002). They presented soft set first application in decision making by representing it in Boolean table and defined its reduct set. Their work of reduct was improved by Chen et al., further improved by Kong et al. and sequentially by Ma et al. for decision making of sub-optimal choices and simplified approaches, respectively (Chen et al. 2005; Kong et al. 2008; Ma et al. 2011). In parallel to these developments, researchers used soft set for handling daily life’s uncertain data issues and applied it in verity of useful applications (Cagman and Enginoglu 2012; Cagman et al. 2011; Çelik and Yamak 2013; Herawan and Deris 2011; Jun et al. 2009; Jun and Park 2008; Kalaichelvi and Malini 2011; Kalayathankal and Singh 2010; Tanay and Kandemir 2011; Xiao et al. 2009; Yuksel et al. 2013). But in some applications, researchers faced problem of incomplete soft set cases with partially missing values. Soft and its related sets data can be missed due to many factors such as improper entry, viral attack, security reasons and errors during data transfer. Incomplete soft sets can be no longer applied in any application or may yield extra-large, very small, unexpected and misleading results, if still applied. Such results, especially a wrong decision making can cause a huge loss to an individual or organizations. For coping with this situation, Zou et al. presented their techniques of weighted-average for calculating decision values and average probability for prediction of missing values in soft set and fuzzy soft set respectively (Zou and Xiao 2008). Qin et al. proposed DFIS where it indicated that data prediction in incomplete soft set is more reliable and accurate if recalculated through association between parameters and they used simple probability for cases having zero or weak association (Qin et al. 2012). Rose et al. also contributed in completion of incomplete soft set using parity bits and aggregate values (Mohd Rose et al. 2011; Rose et al. 2011). Sub-sequentially, Kong et al. (Kong et al. 2014) improved Zou et al. (Zou and Xiao 2008) approach of incomplete soft set by presenting an equivalent probability technique having less complexity and also determining actual missing data instead of only decision values determination. However, in reviewing Kong et al. approach, it still facing inherited shortcomings and low accuracy as compared to DFIS.

In this paper, we compare all exiting approaches in term of accuracy and computational complexity and find DFIS as most suitable among them for predicting missing values in incomplete soft set. We propose an alternative data filling approach for prediction of missing data in soft sets. In summary the contribution of this work is described as follow:

- We propose an alternative data filling approach for prediction of missing data in soft sets (ADFIS). The novelty of ADFIS is that, unlike the previous approach that used probability, we focus more on reliability of association between parameters.
- In contrast to DFIS, we revise association calculating procedure to predict maximum possible number of unknowns through association.
- To validate our work, we perform extensive experiment tests on 04 UCI benchmark and causality workbench lung cancer (LUCAP2) data sets to show the performance of ADFIS.
- We compare the results with other baseline approaches mentioned in the literatures.

### Soft set

Let given *U* be an initial non-empty universal set and *E* be a set of parameters related to *U*. According to Molodtsov (1999), a pair (*F*, *E*) is called soft set over *U* if and only if *F* is mapping from *E* into the set of all subsets of the set *U*. The following example gives us illustration for a soft set.

#### *Example 1*

Suppose *U* = {*h*_1_, *h*_2_, *h*_3_, *h*_4_, *h*_5_} is a set of houses and *E* = {*e*_1_, *e*_2_, *e*_3_, *e*_4_, *e*_5_, *e*_6_} is the set of parameters in relation to each house. Each member of *E* represents cheap, new, wooden, expensive, old and beautiful house, respectively. Let cheap houses are *h*_1_, *h*_3_, *h*_5_, new houses are *h*_1_, *h*_2_, *h*_3_, *h*_4_, wooden houses are *h*_2_, *h*_3_, *h*_4_, expensive houses are *h*_2_, *h*_4_, old house is *h*_5_ and beautiful houses are *h*_1_, *h*_2_, *h*_4_, *h*_5_. Here, the pair (*F*, *E*) describing the attractiveness a soft set given by

$$\begin{aligned} \left( {F,E} \right) & = \left\{ {\left( {e_{1} ,\left\{ {h_{1} ,h_{3} ,h_{5} } \right\}} \right),\,\left( {e_{2} ,\left\{ {h_{1} ,h_{2} ,h_{3} ,h_{4} } \right\}} \right),\,\left( {e_{3} ,\left\{ {h_{2} ,h_{3} ,h_{4} } \right\}} \right)} \right. \\ &\; \quad \left. {\left( {e_{4} ,\left\{ {h_{2} ,h_{4} } \right\}} \right),\left( {e_{5} ,\left\{ {h_{5} } \right\}} \right),\,\left( {e_{6} ,\left\{ {h_{1} ,h_{2} ,h_{4} ,h_{5} } \right\}} \right)} \right\} \\ \end{aligned}$$

### Representation of soft set in tabular form

If *U* is finite non-empty set of objects, *AT* is the non-empty finite set of attributes, $V = \cup V_{r}$ such that *V*_*r*_ is the value domain of attribute and *f* is an information function given by $f:U \times AT \to V_{r}$. Then the quaternion *S* = (*U*, *AT*, *V*_*r*_, *f*) is called an information system (Ma et al. 2011). The soft set (*F*, *E*) in Example 1 is represented in Table 1 i.e. in a Boolean information system.

**Table 1:** Tabular representation of a soft set (*F*, *E*) in a Boolean-valued information system and its decision value

*U*/*E*	*e*_1_	*e*_2_	*e*_3_	*e*_4_	*e*_5_	*e*_6_	*d*_*i*_
*h*_1_	1	1	0	0	0	1	3
*h*_2_	0	1	1	1	0	1	4
*h*_3_	1	1	1	0	0	0	3
*h*_4_	0	1	1	1	0	1	4
*h*_5_	1	0	0	0	1	1	3

In above Table, the objects are represented in rows and parameters in columns. Parameters belonging to a particular object are simply represented by 1 otherwise 0. In soft set-based decision making, the decision value or choice for Mr. Gul among all these houses is given by

$$d_{i} = \sum\limits_{j} {h_{ij} } ,$$

where optimal choice is *max* (*d*_*i*_) and *h*_*ij*_ are the values of elements.

From Table 1, the maximum value is 4 resulted by both houses *h*_2_ and *h*_4_. Hence, either *h*_2_ or *h*_4_ can be his optimal house choice while other houses are sub-optimal options. In the following section, we discuss the incomplete soft set.

### Incomplete soft set

An information system $S^{*} = \left( {U,AT,V_{r} ,f} \right)$ is called incomplete if *f*(*x*_*i*_, *a*_*j*_) is not known, where, *U* = (*x*_1_, *x*_2_, …, *x*_*n*_), *AT* = (*a*_1_, *a*_2_, …, *a*_*m*_), $x_{i} \in U$, *i* = (1, 2, 3, …, *n*) and $a_{j} \in AT$ for *j* = (1, 2, 3, …, *m*). The following example presents an incomplete information system, where unknown entries in the table are represented by symbol “*”. The following example gives us illustration for an incomplete information system representing an incomplete soft set.

#### *Example 2*

Suppose *U* = (*s*_1_, *s*_2_, *s*_3_, …, *s*_8_) is a set of applicants with parameters set *E* = {*e*_1_, *e*_2_, *e*_3_, *e*_4_, *e*_5_, *e*_6_} representing “young age”, “experienced”, “married”, “the highest academic degree is Master”, “studied abroad”, and “the highest academic degree is Doctor”, respectively with its soft set illustration in presented as a Boolean-valued information system in Table 2.

**Table 2:** Representation of incomplete soft set

*U*/*E*	*e*_1_	*e*_2_	*e*_3_	*e*_4_	*e*_5_	*e*_6_
*s*_1_	0	1	1	1	0	0
*s*_2_	0	1	0	0	0	1
*s*_3_	1	0	0	1	0	0
*s*_4_	1	0	$*_{1}$	0	$*_{2}$	1
*s*_5_	0	1	1	0	0	1
*s*_6_	1	0	0	$*_{3}$	0	0
*s*_7_	$*_{4}$	1	1	1	0	0
*s*_8_	0	0	1	0	0	1

From incomplete Boolean Table 2, we know that candidate 4 is young, inexperienced, having Ph.D. as his highest degree, but it is unknown that whether he is married and studied abroad or not. Similarly for candidate 6 and 7, the “highest degree is master” and “young age” values are unknown respectively. Hence it is an incomplete soft set with unknown values represented by $*_{1}$, $*_{2}$, $*_{3}$ and $*_{4}$.

## Related works

In this section, we discuss three of previous soft set-based approaches for handling incomplete data. First we review each of these techniques one by one and then compare them to indicate the most appropriate one for soft set missing data prediction.

### Zou et al. approach

The approach of Zou et al. (Zou and Xiao 2008) has used weighted average technique for decision value calculation of incomplete soft set while incomplete fuzzy soft set’s missing data is predicted through average probability. Here, in relation to our work, we discuss their soft set case only. According to this approach $d_{i} = \sum\nolimits_{i = 1}^{m} {k_{i} c_{i} }$ where *d*_*i*_ is the required decision value *c*_*i*_ is the choice value, *m* is maximum number of choices for same object having missing value and *k*_*i*_ is the weight of choice values. For one missing value, the choice values of an object are only two (0 or 1), hence its respected weights are $k_{1} = \frac{{n_{0} }}{{n_{0} + n_{1} }} = q_{{e_{i} }}$ and $k_{2} = \frac{{n_{1} }}{{n_{1} + n_{0} }} = p_{{e_{i} }}$. For more than one missing values *t* of same object, the choice values increases and its respective weight values are calculated by

$$k = \left\{ {\begin{array}{*{20}l} {\prod\nolimits_{{e \in E_{0}^{*} }} {q_{e} } } \hfill & {x = 0,} \hfill \\ {\sum\nolimits_{{C_{x}^{t} }} {\left( {\left( {\prod\nolimits_{{e_{i} \in E_{1}^{*} }} {p_{{e_{i} }} } } \right)\left( {\prod\nolimits_{{e_{j} \in E_{0}^{*} }} {q_{{e_{j} }} } } \right)} \right)} } \hfill & {0 < x < t,} \hfill \\ {\prod\nolimits_{{e \in E_{1}^{*} }} {p_{e} } } \hfill & {x = t} \hfill \\ \end{array} } \right.$$

where, *x* is the number of 1s in the row, while $E_{1}^{*}$ and $E_{0}^{*}$ are its parameter sets for value 1 and 0 respectively. Using this approach, the decision value in term of candidate’s eligibility for incomplete Table 2 is calculated as explained in related article (Zou and Xiao 2008) and given in Table 3.

**Table 3:** Decision value calculated by Zou et al. technique for incomplete soft set of Example 2

*U*/*E*	*e*_1_	*e*_2_	*e*_3_	*e*_4_	*e*_5_	*e*_6_	*d*_*i*_
*s*_1_	0	1	1	1	0	0	3
*s*_2_	0	1	0	0	0	1	2
*s*_3_	1	0	0	1	0	0	2
*s*_4_	1	0	$*_{1}$	0	$*_{2}$	1	2.57
*s*_5_	0	1	1	0	0	1	3
*s*_6_	1	0	0	$*_{3}$	0	0	1.43
*s*_7_	$*_{4}$	1	1	1	0	0	3.43
*s*_8_	0	0	1	0	0	1	2

### Qin et al. approach

The approach proposed by Qin et al. (Qin et al. 2012) prefers to predict missing value through association between parameters. This association is considered as the first case of their approach. For instance, in Example 1, it is an inconsistent association that an old house can’t be new and cheap can’t be expensive. Similarly, in same example beautiful house is most probably expensive is consistent association. In Example 2, a highest degree can be either master or doctorial indicating inconsistent associations.

Mathematical description of this technique is explained below.

The consistent association between two parameters is found by

1 $$CN_{ij} = \left| {\left\{ {\left. x \right|F_{{e_{i} }} (x) = F_{{e_{j} }} (x),x \in U_{ij} } \right\}} \right|,$$

where *CN*_*ij*_ is the number of elements in column (parameter) *i* having same value to the number of parameter (column) *j*.

Consistent association degree is calculated by

2 $$CD_{ij} = \frac{{CN_{ij} }}{{\left| {U_{ij} } \right|}},$$

where $\left| {U_{ij} } \right|$ is the cardinality (absolute number) of known element’s pairs for parameter *i* and *j*. i.e. *CD*_*ij*_ is the ratio of consistency to number of total elements in columns *i* and *j*.

Similarly, inconsistent association is found as

3 $$IN_{ij} = \left| {\left\{ {\left. x \right|F_{{e_{i} }} (x) \ne F_{{e_{j} }} (x),x \in U_{ij} } \right\}} \right|.$$

And inconsistent association degree is calculated by

4 $$ID_{ij} = \frac{{IN_{ij} }}{{\left| {U_{ij} } \right|}}.$$

To know that whether the association is consistent or inconsistent, net association degree is obtained by

5 $$D_{ij} = \hbox{max} \left\{ {CD_{ij} ,ID_{ij} } \right\}.$$

To find the two parameters having maximum association with each other, the maximal association degree is obtained among the set of all association degrees by

6 $$D_{i} = \hbox{max} \left\{ {D_{ij} } \right\}.$$

As a result, the unknown(s) value $F_{{e_{i} }} (x)$ is predicted as same as the corresponding element(s) *j* (0 for 0 and 1 for 1) if the association is consistent, otherwise it is predicted as a complement of the parameter *j* for inconsistent association.

In second case, when there is weak association between parameters i.e. $\left| {D_{i} } \right| < \lambda$, where λ is a pre-set threshold value. Then, probability for zero and one is calculated as

$$p_{1} = \frac{{n_{1} }}{{n_{1} + n_{0} }}\quad {\text{and}}\quad p_{0} = \frac{{n_{0} }}{{n_{0} + n_{1} }},$$

where *n*_1_ and *n*_0_ are the number of 1s and 0s respectively for the parameter having missing data. As a result, the missing value is put as 1 if *p*_1_ > *p*_*o*_, 0 if *p*_1_ < *p*_*o*_ and either 1 or 0 if *p*_1_ = *p*_*o*_. The following example explains DFIS approach step by step.

#### *Example 3*

Predicting values through DFIS for incomplete case of Example 2. Here the parameters *e*_1_, *e*_3_, *e*_4_ and *e*_5_ have missing data.

*Step 1* Finding consistency *CN*_*ij*_ and inconsistency *IN*_*ij*_.

First we consider parameter 1 with 2: as only *s*_8_ has the same value equal to 0 for both *e*_1_ and *e*_2_, therefore, *CN*_12_ = 1, as the values are not same for all other 6 objects excluding the missing *s*_7_, therefore, *IN*_12_ = 6. Similarly, (*CN*_13_ = 1, *IN*_13_ = 5), (*CN*_14_ = 4, *IN*_14_ = 2), (*CN*_15_ = 4, *IN*_15_ = 2) and (*CN*_16_ = 2, *IN*_16_ = 5).

*Step 2* Calculating ratio of consistency *CD*_*ij*_ and ratio of inconsistency *ID*_*ij*_.

First we need to find cardinality ($\left| {U_{ij} } \right|$) for calculating *CD*_*ij*_ and *ID*_*ij*_. As parameters 1 and 2 have seven complete pairs for all objects except object *s*_7_, therefore, $\left| {U_{12} } \right| = 7$. Similarly, $\left| {U_{13} } \right| = \left| {U_{14} } \right| = \left| {U_{15} } \right| = 6$ and $\left| {U_{16} } \right| = 7$.

Hence, *CD*_12_ = ${{CN_{12} } \mathord{\left/ {\vphantom {{CN_{12} } {\left| {U_{12} } \right|}}} \right. \kern-0pt} {\left| {U_{12} } \right|}}$ = 1/7 = 0.14 and *ID*_12_ = 0.86. Similarly, (*CD*_13_ = 0.16, *ID*_13_ = 0.83), (*CD*_14_ = 0.67, *ID*_14_ = 0.33), (*CD*_15_ = 0.67, *ID*_15_ = 0.33) and (*CD*_16_ = 0.28, *ID*_16_ = 0.83).

*Step 3* Deciding whether association is consistent or inconsistent.

As *D*_*ij*_ = max{*CD*_*ij*_, *ID*_*ij*_}, therefore, *D*_12_ = max{*CD*_12_, *ID*_12_} = max{0.86, 0.14} = 0.86. As the association is inconsistent therefore, minus (−) sign will be used for its indication and differentiation from consistent one i.e. *D*_12_ = −0.86. Similarly, *D*_13_ = −0.83, *D*_14_ = 0.67, *D*_15_ = 0.67 and *D*_16_ = −0.83.

*Step 4* Calculating maximal degree of association.

*D*_*ij*_ is calculated according to step 3 for those parameters having missing values (*e*_1_, *e*_3_, *e*_4_ and *e*_5_) with all other parameters (*e*_1_, *e*_2_, *e*_3_, …, *e*_6_) as presented in Table 4.

**Table 4:** Calculation of *D*_*ij*_

$E^{*} /E$	*e*_1_	*e*_2_	*e*_3_	*e*_4_	*e*_5_	*e*_6_
*e*_1_	–	−0.86	−0.83	0.67	0.67	−0.83
*e*_3_	−0.83	0.71	–	±0.5	−0.67	0.57
*e*_4_	0.67	0.57	±0.5	–	±0.5	−1
*e*_5_	0.67	−0.57	0.57	±0.5	–	0.57

From Table 4, we see that for *e*_1_, *D*_1_ = max{D_12_, D_13_, D_14_, D_15_, D_16_} = max{0.86, 0.83, 0.67, 0.67, 0.83} = −0.86. Similarly, *D*_3_ = −0.83, *D*_4_ = −1 and *D*_3_ = 0.67.

*Step 5* Putting values according to association

We set the threshold λ = 0.85. Only *e*_1_ and *e*_4_ are satisfying the condition to be calculated by association because, $D_{1} = \left| { - 0.86} \right| > \lambda$ and $D_{4} = \left| { - 1} \right| > \lambda$. From Table 4, *e*_1_ has inconsistent association with *e*_2_ and the corresponding element (*u*_72_) of its missing element ($*_{4}$ = *u*_71_) has the value equal to 1 in Table 2. As complement value is assigned in case of inconsistent association, therefore, we put $*_{4}$ = 0. Similarly, we calculate $*_{3}$ = 1.

*Step 6* Calculating probabilities for weak association.

As *D*_3_ and *D*_5_ have smaller values than our fixed threshold λ = 0.85. Therefore, we can’t calculate $*_{1}$ and $*_{2}$ through association, rather we use probability for predicting these values. For *e*_3_ we have *n*_1_ = 4 and *n*_0_ = 3 implies that $p_{1} = \frac{4}{4 + 3} = 0.57$ and $p_{0} = \frac{3}{3 + 4} = 0.43$, as *p*_1_ > *p*_0_, therefore, we put $*_{1} = 1$. Similarly, we calculate $*_{2} = 0$. We obtain a complete Table 5 after putting all predicted values using DFIS in incomplete Table 2.

**Table 5:** Incomplete soft set completed using DFIS, predicted values are shown in italics

*U*/*E*	*e*_1_	*e*_2_	*e*_3_	*e*_4_	*e*_5_	*e*_6_
*s*_1_	0	1	1	1	0	0
*s*_2_	0	1	0	0	0	1
*s*_3_	1	0	0	1	0	0
*s*_4_	1	0	*1*	0	*0*	1
*s*_5_	0	1	1	0	0	1
*s*_6_	1	0	0	*1*	0	0
*s*_7_	*0*	1	1	1	0	0
*s*_8_	0	0	1	0	0	1

### Kong et al. approach

The approach proposed by Kong et al. (Kong et al. 2014) is equivalent to Zou et al. approach (Zou and Xiao 2008) in results but more simplified with respect to complexity. Instead of using weighted-average huge computations, its uses simple probability $p_{{e_{j} }}' = \frac{{n_{1} }}{{n_{1} + n_{0} }}$ for calculating an unknown value, where *n*_1_ and *n*_0_ are the number of 1 and 0 respectively for same parameter. After inserting this value in unknown the decision value is calculated by $d_{i} = \sum\nolimits_{j = 1}^{m} {h_{ij} }$. Using this technique, the incomplete Example 2 gets completed as given in Table 6 along with decision value *d*_*i*_.

**Table 6:** Incomplete soft set of Example 2 after completion and *d*_*i*_ calculation using Kong et al. approach

*U*/*E*	*e*_1_	*e*_2_	*e*_3_	*e*_4_	*e*_5_	*e*_6_	*d*_*i*_
*s*_1_	0	1	1	1	0	0	3
*s*_2_	0	1	0	0	0	1	2
*s*_3_	1	0	0	1	0	0	2
*s*_4_	1	0	$\frac{4}{4 + 3}$	0	$\frac{0}{0 + 7}$	1	2.57
*s*_5_	0	1	1	0	0	1	3
*s*_6_	1	0	0	$\frac{3}{3 + 4}$	0	0	1.43
*s*_7_	$\frac{3}{3 + 4}$	1	1	1	0	0	3.43
*s*_8_	0	0	1	0	0	1	2

### Comparison of previous approaches

As Zou et al. and Kong et al. approaches have approximately same results and Zou et al. approach is compared with DFIS with details (Kong et al. 2014). To conclude, we adopt below associative way for comparing all three previous techniques.

### Zou et al. versus Kong et al

As Zou et al. approach calculates only decision value of incomplete soft set and the missing data remains still missing. While, Kong et al. approach has same results of *d*_*i*_ as that of Zou et al. approach along with assigning a set of values to originally missed information. Secondly, the computational complexity of Kong et al. approach is *O*(*n*^2^) while that of Zou et al. approach is $O\left( {n.2^{n} } \right)$ showing that Kong et al. approach is less complex compare to Zou et al. approach (Kong et al. 2014). Therefore, Kong et al. technique is more appropriate and efficient than Zou et al. approach.

### Kong et al. versus DFIS

As Kong et al. approach works only on probability, ignoring any association between parameters might result probably in different values from actual. Secondly, it predicts missing values in [0, 1] range, while the actual value must be either 0 or 1 in standard soft set (Boolean information system). In contrast, DFIS prefer to predict actual values through association and use probability when the association is not strong. Secondly, in both cases, it calculates binary values maintaining the integrity of standard soft set. Thirdly, compare to Zou et al. results; its decision values results are much closer to actual values as shown in experimental results (Qin et al. 2012). The average of mean absolute percentage error (MAPE) of DFIS is 0.07, while that of Zou et al. approach is 0.11 for all five data sets used in DFIS. If we convert this average of MAPE to percent accuracy of both approaches then the average accuracy of DFIS is 93.17 % while that of Zou et al. approach is 89.12 % in calculating decision values. It is notable that Zou et al. and Kong et al. approaches have same results of decision values (Kong et al. 2014); consequently, the average accuracy of DFIS in decision values comes to be 4.04 % higher than Kong et al. technique. Hence DFIS is more suitable than Kong et al. approach.

In above associative comparison, we showed that Kong et al. technique is better than Zou et al. technique and DFIS is better than Kong et al. technique. Moreover, we calculate the computational complexity of DFIS which consists of below steps.

1. Access whole data set of *m* × *n* size once for getting the number of missing values
2. Compute the degrees of consistencies and inconsistencies of complexity *n*
3. Compute probability of *n* complexity when the association is weak
4. Access once again *m* × *n* table for inserting the computed values

Combining all, results in *m* × *n* + *n* + *n* + *m* × *n* = $2mn + 2n$. Supposing *m* = *n* and considering big O notation, then $2mn + 2n = 2n^{2} + 2n \ge 2n^{2} \ge n^{2}$ for larger values of *n*. Hence, the complexity of DFIS is *O*(*n*^2^), which is equal to the complexity of Kong et al. approach. Therefore, DFIS is most appropriate for missing data prediction in soft set among all three previous approaches. This comparison is summarized in Table 7 as follow:

**Table 7:** Comparison of previous approaches with DFIS

Advantages	Zou et al. (Zou and Xiao 2008)	Kong et al. (Kong et al. 2014)	DFIS (Qin et al. 2012)
Calculates missing value	No	Yes	Yes
Less complexity	No	Yes	Yes
Use association between parameters	No	No	Yes
Calculates binary values (standard soft set)	No	No	Yes
High accuracy	No	No	Yes

Hence, from above associative comparison visualized in Table 7, we conclude that DFIS is more suitable than Zou et al. and Kong et al. approaches for prediction of missing values in soft set. However, in reviewing DFIS, accuracy is still its main problem. Therefore, the following section discusses an alternative data filling approach for prediction of missing data in soft sets, namely ADFIS.

## Alternative approach for data filling of incomplete soft sets

In this section an alternative approach for data filling of incomplete soft sets (ADFIS) is presented. The previous approach DFIS preferred association between parameters to predict missing values than probability and we discussed that association results in more accurate values than probability. But DFIS itself is unable to precisely consider all possible associations for getting more accurate results. In contrast to DFIS, we revise the association calculating method to consider all possible associations precisely and predict maximum possible number of unknowns through it. The novelty of ADFIS is that, it focuses more on reliability of association than DFIS.

For ADFIS, we use Eqs. (1)–(4) to calculate consistent and inconsistent associations and its consistency degrees as DFIS. In case of DFIS, for *n* number of parameters containing missing values, Eq. (5) gives *n* number of *D*_*ij*_s and Eq. (6) is applied separately to each parameter for calculating maximum degree for parameter *i* with parameter *j*. Therefore, Eqs. (5) and (6) are not applied to ADFIS directly. To select one value as the strongest association among all parameters, we use below relation.

7 $$SA_{ij} = \left| {\hbox{max} \left\{ {\hbox{max} \left\{ {CD_{ij} ,ID_{ij} } \right\}} \right\}} \right|,$$

where *CD*_*ij*_, *ID*_*ij*_ are the degrees of consistencies and inconsistencies of each parameter *i* containing missing values with all other parameters *j* and *SA*_*ij*_ is the strongest association among all parameters, between parameter *i* (containing unknown) and (corresponding) parameter *j*. The following definition presents the notion of consistency between two parameters.

### **Definition 1**

Two parameters *e*_*i*_ and *e*_*j*_ are said to be consistent $e_{i} \, \Leftrightarrow e_{j}$ with each other if there is strongest association between them. i.e. *SA*_*ij*_ ≥ *λ* and max{*CD*_*ij*_, *ID*_*ij*_} = *CD*_*ij*_, where *λ* is a pre-set threshold values (for more details, see “Discussions”).

From Definition 1, it can be seen that if two parameters are consistent to each other, then its corresponding elements are also consistent with each other. If $e_{i} \Leftrightarrow e_{j}$ then $F(e)_{ni} \Leftrightarrow F(e)_{nj}$, if $F(e)_{ni} = *$ then

8 $$F(e)_{ni} = F(e)_{nj}$$

where, * is unknown and *n* is the object position (row) of parameter value *F*(*e*). The following definition presents the notion of inconsistency between two parameters.

### **Definition 2**

Two parameters *e*_*i*_ and *e*_*j*_ are said to be inconsistent $e_{i}\;{ \Rrightarrow }\;e_{j}$ with each other if there is strongest inconsistent association between them. i.e. *SA*_*ij*_ ≥ *λ* and max{*CD*_*ij*_, *ID*_*ij*_} = *ID*_*ij*_.

From Definition 2, it can be seen that if two parameters are inconsistent to each other, then its corresponding elements are also inconsistent with each other. If $e_{i}\; { \Rrightarrow }\;e_{j}$ then $F(e)_{ni}\;{ \Rrightarrow}\;F(e)_{nj}$, if $F(e)_{ni} = *$ then

9 $$F(e)_{ni} = 1 - F(e)_{nj}$$

where, * is unknown and *n* is the object position (row) of parameter value *F*(*e*). The following definition presents the notion of non-association between two parameters.

### **Definition 3**

Two parameters *e*_*i*_ and *e*_*j*_ are said to be non-associated $e_{i} { \nLeftrightarrow }e_{j}$ if there exist no strongest association between them i.e. *SA*_*ij*_ < *λ.*

From Definitions 1–3, we derive our proposed algorithm of ADFIS as described below.
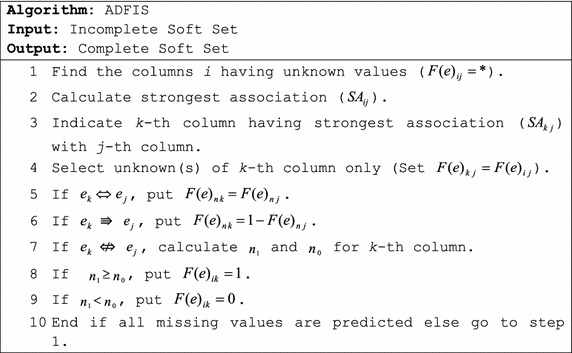


From above algorithm, the ADFIS firstly calculates the unknown(s) of the column having greatest association than all other columns among whole table. Before proceeding to further prediction, it inserts the recently calculated value(s) having strongest association in incomplete table. In next step, it again calculates association among parameters of whole table with consideration of the weight of recently inserted (most reliable) value(s) and finds strongest association again. The process of finding strongest association and predicting unknowns is repeated until all unknown data is filled or the condition of threshold disqualifies. In case of weak association, ADFIS uses simple comparison of *n*_1_ and *n*_0_ instead of calculating *p*_1_ and *p*_0_.

The main difference between DFIS and ADFIS is that, DFIS calculates association among all parameters only once and decides on its base but ADFIS calculates it again and again after inserting the unknown value in one column being calculated through strongest association.

ADFIS is further explained for understanding and comparison with DFIS in Example 4 with same incomplete case of Example 2.

### *Example 4*

Prediction of unknowns for incomplete soft set case Example 2 through ADFIS. Consider Example 2 and Table 2, for same case and same threshold value (λ = 0.85).

*Step 1* We construct Table 8 containing the values of max{*CD*_*ij*_, *ID*_*ij*_}.

**Table 8:** max{*CD*_*ij*_, *ID*_*ij*_} − 1

$E^{*} /E$	*e*_1_	*e*_2_	*e*_3_	*e*_4_	*e*_5_	*e*_6_
*e*_1_	–	−0.86	−0.83	0.67	0.67	−0.83
*e*_3_	−0.83	0.71	–	±0.5	−0.67	0.57
*e*_4_	0.67	0.57	±0.5	–	±0.5	−1
*e*_5_	0.67	−0.57	0.57	±0.5	–	0.57

From Table 8, according to Eq. (7) *SA*_46_ = 1, for parameter 4 with parameter 6.

As *SA*_*ij*_ > λ and max{*CD*_*ij*_, *ID*_*ij*_} = *ID*_*ij*_, definition 2 satisfies, therefore, $e_{4}\;{ \Rrightarrow }\;e_{6}$ and $F(e)_{64}\;{ \Rrightarrow }\;F(e)_{66}$. In Table 2, $F(e)_{64} \, = *_{3}$ hence, we can put $F(e)_{64} = 1-F(e)_{66}$ according to Eq. (9). As *F*(*e*)_66_ = 0 in Table 2, we calculate $F(e)_{64} = 1 - 0 = 1$. Hence we obtain $*_{3} = 1$. After putting this value, we get Table 9 as an updated case of incomplete data.

**Table 9:** Incomplete case after inserting first calculated unknown ($*_{3}$) through strongest association

*U*/*E*	*e*_1_	*e*_2_	*e*_3_	*e*_4_	*e*_5_	*e*_6_
*s*_1_	0	1	1	1	0	0
*s*_2_	0	1	0	0	0	1
*s*_3_	1	0	0	1	0	0
*s*_4_	1	0	$\mathop *\nolimits_{1}$	0	$\mathop *\nolimits_{2}$	1
*s*_5_	0	1	1	0	0	1
*s*_6_	1	0	0	*1*	0	0
*s*_7_	$\mathop *\nolimits_{4}$	1	1	1	0	0
*s*_8_	0	0	1	0	0	1

*Step 2* Including the weight of recently calculated $*_{3}$ in Table 9, we calculate Table 10 containing the new values of max{*CD*_*ij*_, *ID*_*ij*_}.

**Table 10:** max{*CD*_*ij*_, *ID*_*ij*_} − 2 for updated Table 9

*D*_*ij*_	*e*_1_	*e*_2_	*e*_3_	*e*_4_	*e*_5_	*e*_6_
*e*_1_	–	−0.86	−0.83	0.71	0.57	−0.71
*e*_3_	−0.83	0.71	–	−0.57	−0.57	0.57
*e*_5_	0.57	−0.57	−0.57	−0.57	–	0.57

In Table 10, the strongest association is that of *e*_1_ with *e*_2_, *SA*_12_ = |−0.86| > λ, similar to step 1, we put $*_{4} = 0$ and obtain updated Table 11.

**Table 11:** Incomplete case after putting values of 1st and 2nd unknowns $*_{3}$ and $*_{4}$

*U*/*E*	*e*_1_	*e*_2_	*e*_3_	*e*_4_	*e*_5_	*e*_6_
*s*_1_	0	1	1	1	0	0
*s*_2_	0	1	0	0	0	1
*s*_3_	1	0	0	1	0	0
*s*_4_	1	0	$\mathop *\nolimits_{1}$	0	$\mathop *\nolimits_{2}$	1
*s*_5_	0	1	1	0	0	1
*s*_6_	1	0	0	*1*	0	0
*s*_7_	*0*	1	1	1	0	0
*s*_8_	0	0	1	0	0	1

*Step 3* Based on updated Table 11, we recalculate max{*CD*_*ij*_, *ID*_*ij*_} in Table 12 as follow.

**Table 12:** Calculation of max{*CD*_*ij*_, *ID*_*ij*_} − 3 for updated Table 11

$E^{*} /E$	*e*_1_	*e*_2_	*e*_3_	*e*_4_	*e*_5_	*e*_6_
*e*_3_	−0.86	0.71	–	−0.57	−0.57	0.57
*e*_5_	0.71	−0.57	−0.57	−0.57	–	0.57

It can be observed from Table 12 that *SA*_31_ = |−0.86| > λ also entered into defined threshold range of association and we put $*_{1} = 0$ getting updated incomplete case in Table 13.

**Table 13:** After putting value of $*_{1} ,*_{3}$ and $*_{4}$

*U*/*E*	*e*_1_	*E*_2_	*E*_3_	*E*_4_	*e*_5_	*e*_6_
*s*_1_	0	1	1	1	0	0
*s*_2_	0	1	0	0	0	1
*s*_3_	1	0	0	1	0	0
*s*_4_	1	0	*0*	0	$\mathop *\nolimits_{2}$	1
*s*_5_	0	1	1	0	0	1
*s*_6_	1	0	0	*1*	0	0
*s*_7_	*0*	1	1	1	0	0
*s*_8_	0	0	1	0	0	1

*Step 4* The value of max{*CD*_*ij*_, *ID*_*ij*_} for Table 13 is recalculated in Table 14 as follow:

**Table 14:** Calculation of max{*CD*_*ij*_, *ID*_*ij*_} − 4 for updated Incomplete Table 13

$E^{*} /E$	*e*_1_	*e*_2_	*e*_3_	*e*_4_	*e*_5_	*e*_6_
*e*_5_	0.71	−0.57	−0.57	−0.57	–	0.57

As *SA*_51_ = 0.71 in Table 14 means $e_{5}\;{ \nLeftrightarrow }\;e_{1}$ therefore, $*_{2}$ cannot be calculated through association for λ = 0.85. This case is falling under definition 3 and we use probability for it. We see from Table 13, that for *e*_5_, *n*_1_ = 0 and *n*_0_ = 7. As *n*_0_ > *n*_1_ therefore, we put $*_{2} = 0$. Hence, using ADFIS, we obtained all missing values in complete Table 15.

**Table 15:** Completed soft set using ADFIS

*U*/*E*	*e*_1_	*e*_2_	*e*_3_	*e*_4_	*e*_5_	*e*_6_
*s*_1_	0	1	1	1	0	0
*s*_2_	0	1	0	0	0	1
*s*_3_	1	0	0	1	0	0
*s*_4_	1	0	*0*	0	*0*	1
*s*_5_	0	1	1	0	0	1
*s*_6_	1	0	0	*1*	0	0
*s*_7_	*0*	1	1	1	0	0
*s*_8_	0	0	1	0	0	1

## Results and discussion

In this section we discuss the improvement in accuracy of the ADFIS. Firstly, we discuss our incomplete case in Example 2 with prediction results by DFIS and ADFIS from Table 5 and Table 15, respectively. Then, we present the results obtained from DFIS and ADFIS for four UCI benchmark datasets Causality workbench LUCAP2 data set. Some important discussions are provided after the results presentations and shortcomings of ADFIS are also discussed at the end of this section.

### Incomplete soft set of Example 2

Refer to comparison Table 16, all values predicted through DFIS are same as ADFIS except $*_{1}$, although the threshold is same for both approaches. $*_{1}$ got neither only complemented value for both techniques but also calculated through different ways i.e. through association in ADFIS and probability through DFIS. The DFIS proves that association is more reliable than probability; therefore we claim that the value of $*_{1}$ calculated as 0 using association by ADFIS is more accurate than predicted as 1 by DFIS using probability.

**Table 16:** Comparison of DFIS and ADFIS predicted values for incomplete case of Example 2

Unknown	Predicted results through			
	DFIS		ADFIS	
	Value	Using	Value	Using
$\mathop *\nolimits_{1}$	*1*	*Probability*	*0*	*Association*
$\mathop *\nolimits_{2}$	0	Probability	0	Probability
$\mathop *\nolimits_{3}$	1	Association	1	Association
$\mathop *\nolimits_{4}$	0	Association	0	Association

**Fig. 1:**
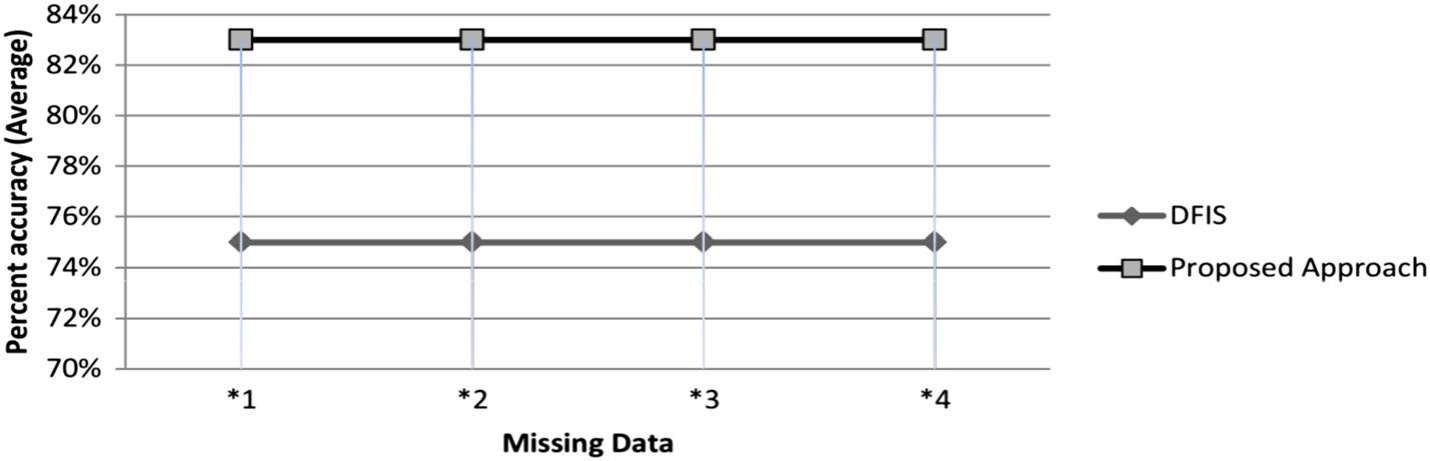
Performance comparison of DFIS and ADFIS for incomplete case of Example 2

Suppose an unknown predicted though association has 90 % accuracy and that predicted through probability has 60 %. Then the average accuracy of DFIS is 75 % while that of ADFIS is 83 % for this case as shown through graph in Fig. 1.

### UCI benchmark data sets

Similar to DFIS (Qin et al. 2012), we tested DFIS and ADFIS for four data sets from UCI benchmark database (UCI Machine Learning Repository 2013).

**Fig. 2:**
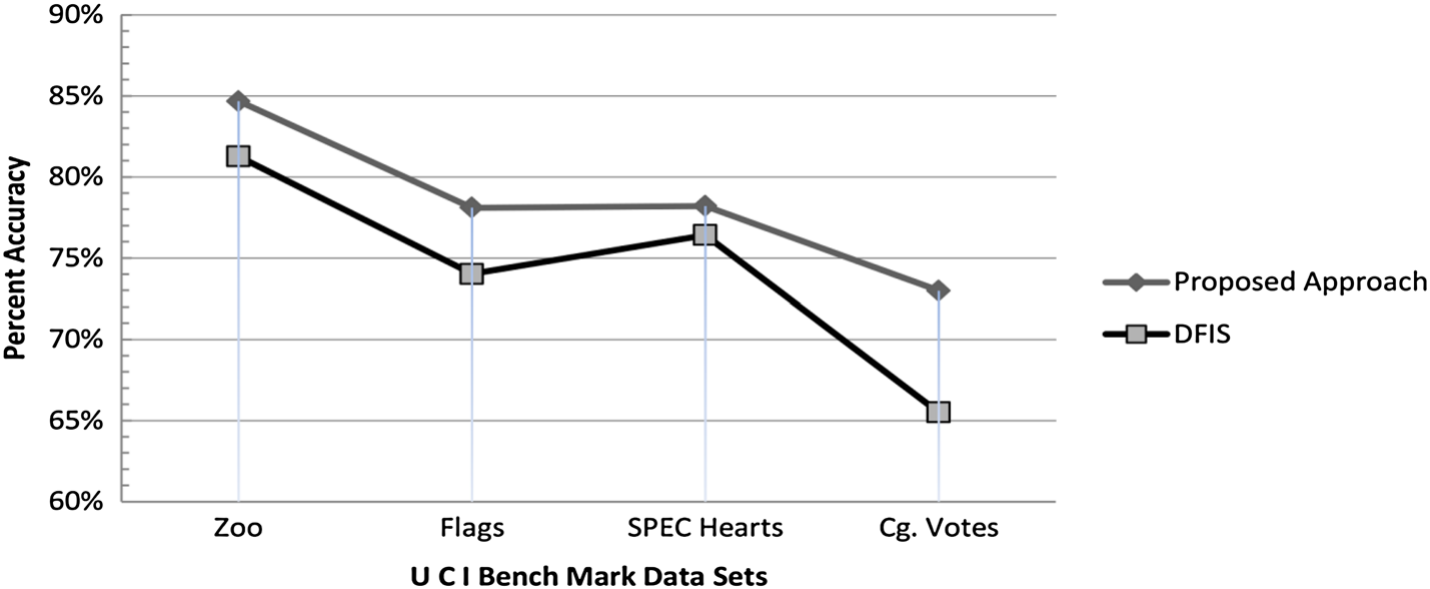
Average accuracy performance comparison of ADFIS and DFIS for UCI benchmark data sets

We randomly deleted 30–600 entries ten times from Zoo, Flags, Congressional votes and SPECT hearts data sets and re-calculated it using both approaches by implementing both algorithms in Matlab. We found that average accuracy of DFIS is 74.30 % while that of ADFIS is 78.49 % i.e. ADFIS performs 4.19 % better than DFIS. Average performance graph is shown Fig. 2. Now we discuss experimental results of each data set one by one.

### Zoo data set

**Fig. 3:**
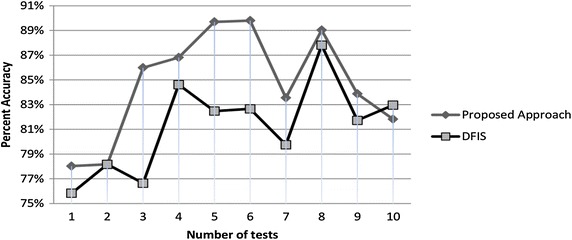
Percentage prediction accuracy for zoo data set

Zoo data set contains 101 types of different animals with their 18 different features like presence of feather, teeth, backbone and hair. We selected only 15 parameters having Boolean values and randomly deleted ten times the number of values 91, 87, 107, 91, 97, 98, 79, 82, 93 and 88 from it. All deleted values are recalculated using both (DFIS and ADFIS) approaches. Percent accuracy graph of these results is given in Fig. 3.

Average performance of DFIS’s accuracy is 81.26 % while that of ADFIS is 84.67 % i.e. ADFIS performs 3.41 % accurate than DFIS for Zoo data set.

### Flags data set

**Fig. 4:**
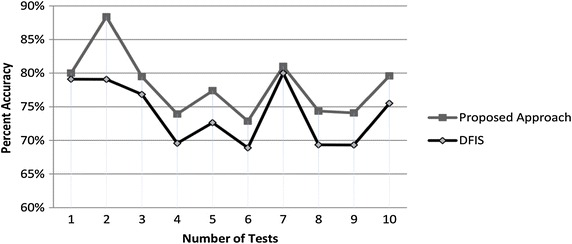
Prediction accuracy percentage of flags data set

Flags dataset contains national flags description of 128 countries with 28 parameters. Out of all only 13 parameters are Boolean which are selected for our testing purpose. Accuracy graph for randomly deleted number of values 110, 43, 151, 92, 84, 151, 200, 538, 189 and 49 is given in Fig. 4 for flag data set. Performance of ADFIS is 4.08 % better than DFIS as DFIS average accuracy is 74.02 % while that of ADFIS is 78.10 %.

### SPECT hearts data set

**Fig. 5:**
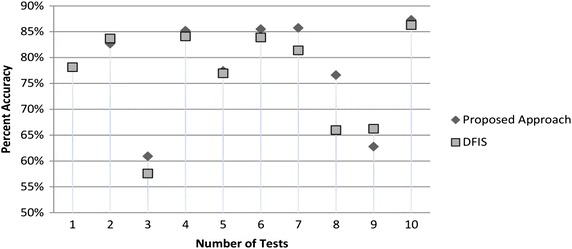
Percentage of accuracy graph of SPECT hearts dataset

SPECT hearts is training data set containing images of SPECT abbreviated from Single Proton Emission Computed Tomography. The data base consists of 80 patients with 22 Boolean valued attributes. Numbers of values randomly deleted are 32, 98, 450, 182, 230, 62, 161, 47, 290 and 102. Percent performance graph is shown in Fig. 5.

Average accuracy of DFIS is 76.41 % while that of ADFIS is 78.20 %. Hence ADFIS performs 1.80 % better than DFIS for SPECT hearts data set.

### Congressional votes data set

This data set contains voting record of US congress members of 1984. 435 members had contested their votes in yes or no regarding 16 issues out of which only 230 members votes are completed. We selected these completed votes only for testing purpose and deleted randomly 161, 435, 122, 98, 263, 239, 205, 291, 424 and 136 values from this data set. After recalculating it though both approaches we found that DFIS average accuracy is 65.50 % while ADFIS has 72.98 % accuracy.

**Fig. 6:**
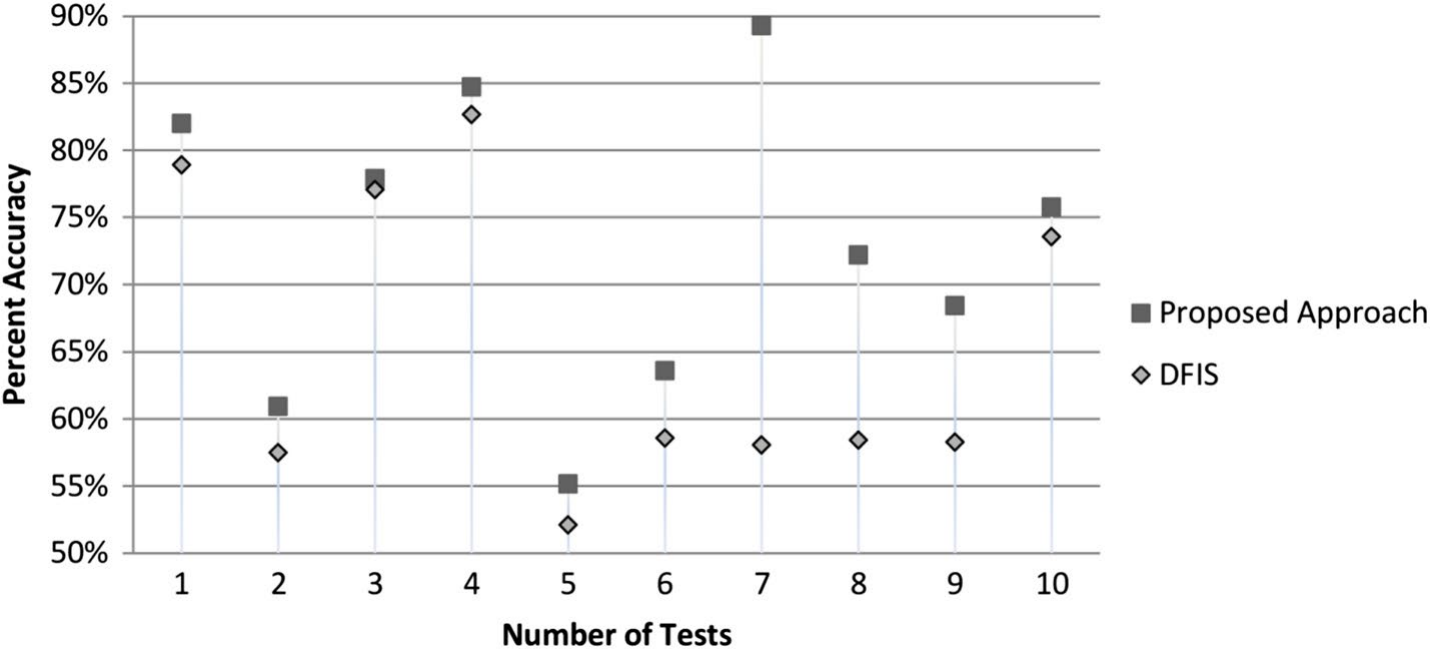
Percent accuracy graph of congressional votes dataset

Average performance of ADFIS is 7.84 % better than DFIS for this data set. Performance graph of ADFIS vs DFIS is plotted in Fig. 6.

### Causality workbench LUCAP2 data set

**Fig. 7:**
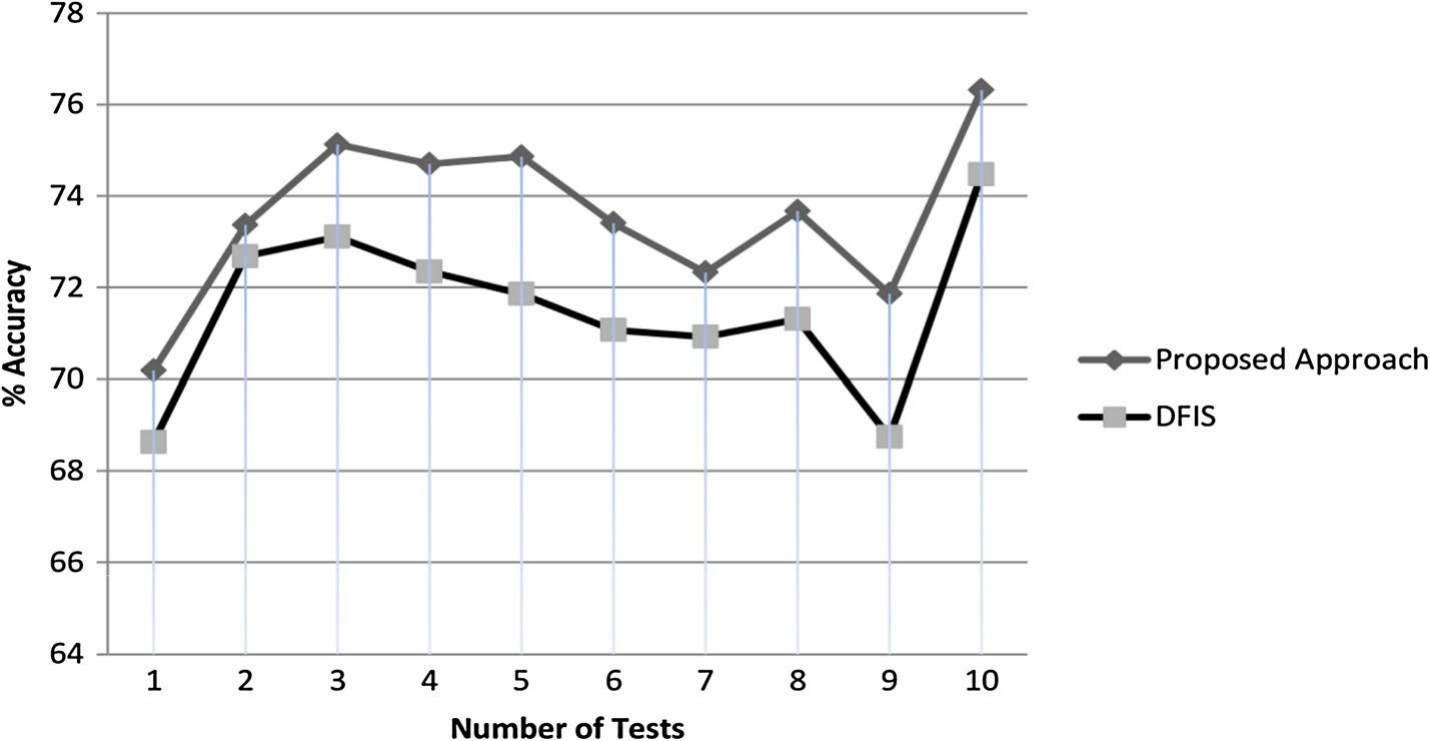
Percent accuracy graph of LUCAP2 dataset

Lung Cancer set with Probes (LUCAP) (Causality Workbench 2013) is an online data set containing Boolean valued artificially generated data by causal Bayesian networks. There are ten thousand imaginary objects (patients) with 143 features (symptoms) like Coughing, Fatigue, Yellow Fingers, Anxiety, Allergy, Attention Disorder and Smoking. Out of 10,000 we selected only first 1000 with all 143 parameters for our testing purpose. We randomly deleted 322, 2354, 1190, 2083, 1432, 1158, 5413, 2457, 899 and 760 number of values and recalculated it through DFIS and ADFIS. We found that for 1807 average unknowns, DFIS calculated 1294, while ADFIS calculated 1328 accurate values. Hence the average performance of ADFIS is 1.89 % better than DFIS for this data set. Percent accuracy graph of DFIS versus ADFIS for LUCP2 data set is given in Fig. 7.

In summary, the overall comparison results are given in the following Table 17.

**Table 17:** Overall accuracy comparison

Data sets	DFIS (%)	ADFIS (%)	Improvement (%)
Example 2	75.00	83.00	8.00
Zoo data set	81.26	84.67	3.41
Flags data set	74.02	78.10	4.08
SPECT hearts data set	76.41	78.20	1.79
Congressional votes data set	65.50	72.98	7.48
LUCAP2 data set	71.61	73.49	1.89
Average			4.44

From Table 17, we can conclude that the ADFIS performs up to 4.4 % better as compared to DFIS.

## Discussions

In this sub-section we discuss some important queries that are raised regarding the threshold (λ), its function, range and suitable values. We also discuss the precise theoretical difference between DFIS and ADFIS, validation of proposed method and performance evaluation.

The threshold lambda (λ) is a filter that can be set according to the requirements of individuals in getting weak or strong associations. Closer the value of λ to 1 result in more reliable association and closer the value to zero might result in selecting weaker associations. To select more than 50 % associational results, the lambda must be fixed to 0.5 or above. In our incomplete case of example 2 we have kept the threshold λ = 0.85 to select only the parameters associations having minimum 85 % similarity between them and the unknowns of parameters having less than 85 % similarity are calculated through probability in DFIS while one of them ($\mathop *\nolimits_{1}$) enters to the threshold range in ADFIS case. This reveals the core difference between DFIS and ADFIS. DFIS calculates all associations once for whole data set and assigns missing values according to it. We notice that those parameters satisfying the threshold can be further categorized in less and more stronger association in the range between threshold and 1. Two parameters might have marginal similarity of 85 % while another set of two may have stronger similarity as 90 % or even 100 %. DFIS treat them all as same for finding missing values, while we calculate the unknown first through the strongest among them and utilize it for its role in upcoming calculations. This way, some of the unknowns that are calculated through probability enters association range and get more probable accurate results, as calculating unknowns through association is more reliable than probability (Qin et al. 2012). The results of DFIS are validated by calculating its decision values and comparing its MAPE with that of Zou et al. approach. As Zou et al. approach does not calculate missing values; therefore DFIS used indirect method of validation. But in our case, both DFIS and ADFIS calculate actual missing values and we do not need to validate it through indirect decision values. So, we use direct method of comparing both techniques’ actual results with original and the more accuracy of ADFIS validates its better performance.

### Weaknesses of the ADFIS

Apart from improved accuracy, there are two main limitations of ADFIS compare to DFIS.

#### Incorrect results rare cases

Sometimes the strongest association becomes false because of too much missing values or no real association existence. In this case, if missing values calculated in first step of ADFIS are incorrect then it affects the result of calculated values in next steps as well. This case can be viewed in the 2nd and 9th test result of SPECT Hearts data set graph where DFIS has high accuracy than ADFIS.

#### High computational complexity

High computational complexity of ADFIS compare to DFIS is obvious. DFIS access a data set of *m* × *n* size once for finding association while ADFIS (*m* × *n*)^2^ times during its execution. Complexity of ADFIS is DFIS times more than that of DFIS.

## Conclusion

In this paper, we have discussed three previous approaches for prediction of incomplete soft set and pointed out DFIS as most suitable among them. We have presented an alternative approach of data filling for incomplete soft set (ADFIS) for the purpose of accuracy improvement. We have re-arranged the process of DFIS, therefore the maximum possible number of unknowns in incomplete soft set can be predicted through association between parameters. We have presented a modified algorithm and explain our ADFIS with the help of an example as a proof of concept. We have also compared the results of ADFIS with the existing DFIS approach after implementing both in Matlab for four UCI benchmark data sets and Causality workbench lung cancer data set (LUCAP2) and shared the average results of both approaches in the form of graphs. ADFIS has improved the percentage of accuracy of predicted unknowns by 4.44 % average as compared to DFIS for all 5 data sets. We mentioned two main snags of ADFIS i.e. rare cases wrong values prediction and high computational complexity which can be resolved in its future work.
